# The mitochondrial genome of *Culter oxycephaloides* (Cypriniformes, Cyprinidae)

**DOI:** 10.1080/23802359.2017.1383196

**Published:** 2019-07-12

**Authors:** Huijuan Chen, Dengqiang Wang, Xinbin Duan, Shaoping Liu, Daqing Chen

**Affiliations:** a Yangtze River Fisheries Research Institute, Chinese Academy of Fishery Sciences, Wuhan, China;; b College of Animal Science and Technology, Southwest University, Chongqing, China

**Keywords:** *Culter oxycephaloides*, Cypriniformes, mitogenome

## Abstract

In the present study, the complete mitogenome sequence of *Culter oxycephaloides* was determined using PCR amplification and DNA sequencing, which contains 13 protein-coding genes, 22 tRNA genes, 2 rRNA genes, and a non-coding control region with the total length of 16,619 bp. Except for eight tRNA and ND6 genes, all other mitochondrial genes are encoded on the heavy strand. The codon usage followed the typical vertebrate mitochondrial pattern (ATG or GTG for start codon and TAA or TAG for stop codon). There are 12 regions of gene share totalling 55 bp and 11 intergenic spacer regions totalling 84 bp. The complete mitochondrial genome sequence is useful for phylogenetic analysis and studies of population genetics of *C. oxycephaloides*.

Here, one *Culter oxycephaloides* was caught in Shashi section of middle reach in Yangtze River, Hubei province in China. Total genomic DNA from fin tissue was extracted using a modified ammonium acetate precipitation protocol (Nicholls et al. 2000) and stored at −20 °C. The rest of the specimens were kept in a laboratory of Yangtze River Fisheries Research Institute, Chinese Academy of Fishery Sciences. We amplified and sequenced the whole mitochondrial genome sequence of *C. oxycephaloides* with 16 pairs of primers designed in accordance with equivalent mitochondrial genome sequences of 30 species from Cypriniformes (Wang et al. 2011). The DNA sequence with a Web-based tool DOGMA (Wyman et al. 2004) was annotated, which has ever been used in many previous studies. Here we presented the complete mitochondrial genome of *C. oxycephaloides* that was deposited in GenBank with the accession number KY404014.

The complete mitochondrial genome of *C. oxycephaloides* was found to be similar to that of most other vertebrates (Huang et al. 2012). It was sequenced and determined to be 16,619 bp in length, including 13 typical vertebrate protein-coding genes, 22 transfer RNA genes, 2 ribosomal RNA genes, and a control region. The majority of the mitochondrial genes of *C. oxycephaloides* are encoded on heavy strand (H-strand) except for ND6 and eight tRNA genes(tRNA-Gln, Ala, Asn, Cys, Tyr, Ser (UCN), Glu, and Pro), which are encoded on light strand (L-strand). The base composition of H-strand was as follows 31.3% A, 27.88% C, 24.75% T, 16.07% G and AT content was 56.06%. The relative order of nucleotide composition matches with the nucleotide pattern of other cyprinids in the order: A > C > T > G (Wang 2008). The GC content of cyprinid mitochondrial genomes is always lower than AT content and this is also evident in other teleost species (Yue et al. 2006). Of the 13 protein-coding genes, gene overlaps can be observed between four pairs of the contiguous genes, ATP8–ATP6, ATP6–COIII, ND4L–ND4, and ND5–ND6, and they overlap by seven, one, seven, and four nucleotides, respectively and 84 bp intergenic nucleotides in the whole genome, ranging from 1 to 31bp. All 13 protein-coding genes of *C. oxycephaloides* share the start codon ATG, except for COI, which begins with GTG. Open reading-frames of five protein-coding genes end with TAA and one protein-coding genes end with TAG, while, the others with incomplete stop codons, TA- (COIII, ND4) or T- (ND2, COII, ND3, ND6 and Cyt b). To further validate the new sequences, MEGA 5 (Tamura et al. 2011) was used to produce the phylogenetic tree of *C. oxycephaloides* and all other *Culter* sequences in NCBI based on the Neighbour-Joining method (Figure 1), with *Ancherythroculter kurematsui* as an outgroup. As expceted, *C. oxycephaloides* clustered with the other two *Culter* species, *C. alburnus* and *C. recurviceps*, and with *Ancherythroculter kurematsui* on different branches.

**Figure 1.**
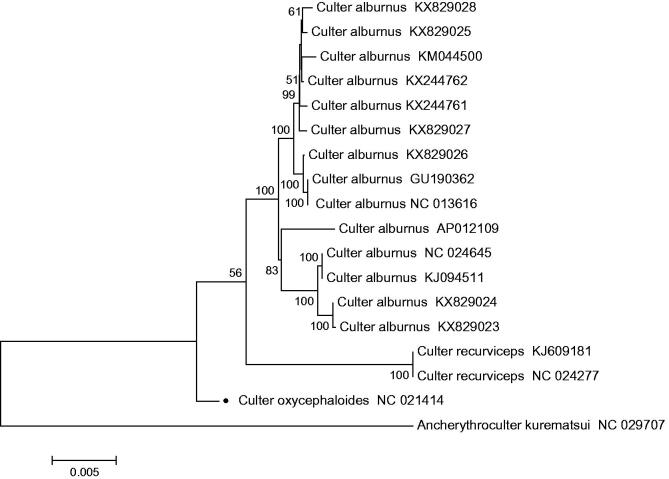
N-J phylogenetic tree of *C. oxycephaloides* and all other *Culter* sequences in NCBI, with *Ancherythroculter kurematsui* as an outgroup. The round dot indicate the individual sampled in this study.

In this study, we reported the complete mitochondrial genome (mitogenome) of *C. oxycephaloides* and hope it may play important roles in the studies of population genetics and phylogenetic analysis of this species.
